# Structural potential of the 5′ noncoding regions of the mRNAs encoding p53 isoforms

**DOI:** 10.3389/abp.2026.15861

**Published:** 2026-02-19

**Authors:** Mariola Dutkiewicz, Paulina Zydowicz-Machtel

**Affiliations:** Institute of Bioorganic Chemistry, Polish Academy of Sciences, Poznań, Poland

**Keywords:** mRNA structure, p53 isoforms, RNA interactions, RNA structure, translation regulation

## Abstract

Famous for its nickname “guardian of the genome,” the p53 protein acts, among other things, as a transcription factor in the form of a tetramer, which may consist of different types of p53 isoforms. They differ in length and content of specific domains that are responsible for their functions. The way this factor acts, sometimes opposite to what we would expect from the main protein isoform, depends on which isoforms form the tetramer. There are over a dozen isoforms of the human p53 protein encoded by a single gene, thanks to the use of different transcriptional promoters (DNA level), alternative splicing (pre-mRNA level), and different translation initiation sites (mRNA level). *In vitro* studies have demonstrated that the use of different translation initiation sites on full-length p53FL mRNA is possible due to specific RNA structures, and that these structures are also responsible for the rate and efficiency of target protein isoform formation. This affects the proportions between the different p53 isoforms present in the cell at a given moment and, consequently, the further fate of the cell. This paper summarizes the knowledge about the importance of the RNA structure (I-III order) of individual p53 transcripts for the fate of the cell and the organism.

## Introduction

The TP53 gene is highly conserved among eukaryotic organisms. In humans, it is located on chromosome 17 (17p13.1) (Lane et al., 2010) and comprises approximately 20,000 base pairs, consisting of 11 exons and 10 introns. The p53 protein is recognized as a crucial tumor suppressor, playing a vital role in regulating key cell cycle processes and responding to cellular stress (Lane and Crawford, 1979; Linzer and Levine, 1979; Kastan et al., 1991). One of the effects of mutations in the TP53 gene is the abnormal expression of its protein, which leads to disruption of the cell cycle and, consequently, to the development of neoplasia. Mutations in the TP53 gene have been found in over 50% of human cancers (Lane and Levine, 2010). It acts primarily as a transcription factor in the form of a tetramer (Khosravi et al., 1999; Ashcroft et al., 1999), stimulating the expression of genes responsible for DNA repair or apoptosis (Kastan et al., 1991).

The 5′ cap-dependent translation initiation process is the most efficient mechanism of protein synthesis for most mRNA transcripts present in the cell. However, the efficiency of this process may be reduced or abolished under conditions of cellular stress, when most of the cell’s vital functions are limited and the action of translation initiation factors, such as eIF4F or eIF2α, is blocked. In such a situation, it is possible to bypass initiation involving the 5′ cap and initiate translation using specialized structures called internal ribosome entry sites (IRES), which recruit a small ribosome subunit to the start codon (Pelletier and Sonenberg, 1988; Spriggs et al., 2008). Thanks to the IRES structure, it is possible to maintain the continuous synthesis of some critical proteins, regardless of unfavorable physiological conditions within the cell. These proteins regulate the cell cycle, apoptosis, cell response to stress conditions, or the process of carcinogenesis. It has been estimated that approximately 10%–15% of cellular transcripts have the ability to translate independently of the cap (Lacerda et al., 2017; Marques et al., 2022). Among them are proteins that are crucial for sustaining life, such as p53 (Ray et al., 2006; Candeias et al., 2006; Harris et al., 2018). This strategy is also employed by some viruses, which, after blocking the cap-dependent mechanism — e.g., by deactivating certain translation factors and utilizing highly efficient IRES structures — redirect the cellular protein biosynthesis machinery to meet their own needs (Pelletier and Sonenberg, 1988; Dutkiewicz et al., 2016). There are also other ways of cap-independent translation initiation, e.g., leaky scanning or re-initiation (Merrick WC, 2004).

## p53 isoforms and mechanisms involved in their generation

**FIGURE 1:**
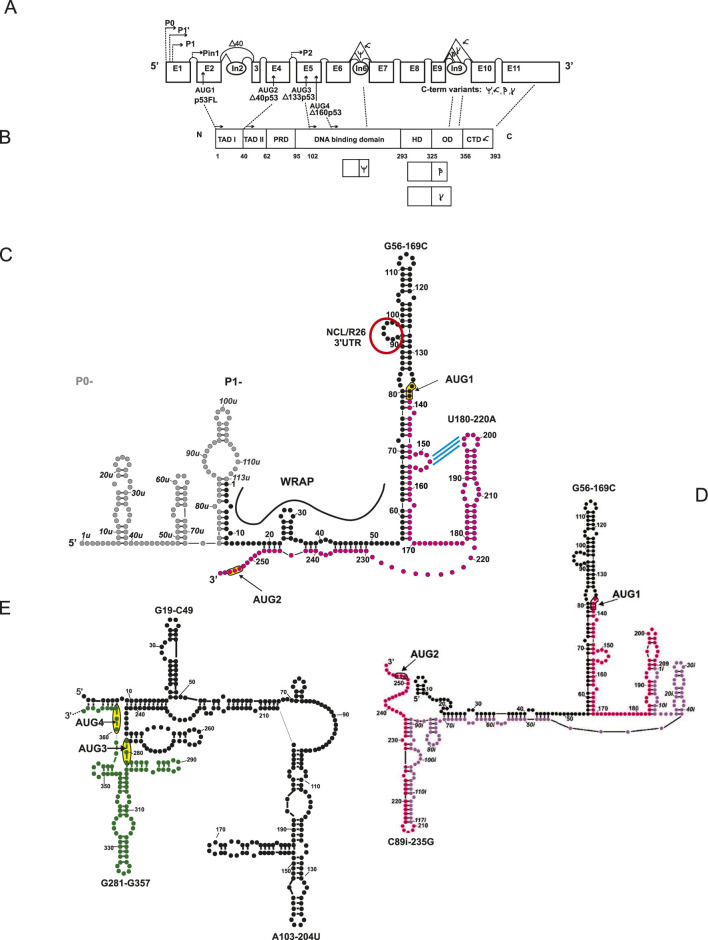
Schematic structure representation of the pre-mRNA **(A)**, protein **(B)**, and 5′ terminal region of mRNA of p53 isoforms **(C–E)**. RNA secondary structure model of the 5′ terminal regions of mRNA for: p53 and Δ40p53 **(C)**, gray circles – sequence between transcription initiation sites P0 and P1, present only in mRNA initiated at P0, black circles – 5′ untranslated region -5′UTR, magenta circles – partial coding sequence; red ring – interaction site with nucleolin (NCL), R26 protein and 3′UTR, blue stripes – proposed interactions within a potential riboswitch mode; RNA secondary structure model of the 5′ terminal region of mRNA for Δ40p53, version with intron 2 **(D)**, lilac circles – intron 2 sequence; RNA secondary structure model of the 5′ terminal regions of mRNA encoding Δ133p53 and Δ160p53 **(E)** black circles – untranslated sequence between P2 (transcription initiation site 2) and AUG3 (translation initiation site 3), includes sequences from intron 4 and exon 4, green circles – partial coding sequence of Δ133p53.

In the 1980s, it was observed that shortened mRNA variants can be produced from the TP53 gene, which encodes different proteins (reviewed in Marcel et al., 2011a). This led to the hypothesis that there are several isoforms of the p53 protein. Subsequent years of research on the TP53 gene expression have shown that, as a result of the use of different transcriptional promoters, alternative splicing, and different translation initiation sites, it is possible to synthesize many isoforms of the p53 protein (Khoury and Bourdon, 2010) (Figure 1; Table 1).

**TABLE 1:** Structure, mechanism of generation and function of the p53 isoforms.

p53 isoform name	mRNA generation	I-, II-, III- RNA structure and RNA-RNA interactions	protein translation mechanism, RNA-protein interactions	protein function
p53FL/p53α/main isoform/full-length protein	transcription from promoter P1 (cancer cells), P0 or P’ (healthy cells)	full-length mRNA: contains all exons, classic 5′UTR and 3′UTR partial secondary structure model has been proposed (Figure 1C; Błaszczyk and Ciesiołka 2011) long-range RNA-protein-RNA interaction with its own 3′UTR (Kiliszek et al., 2023) interaction with antisense transcript WRAPα (Mahmoudi et al., 2009)	classic translation initiation (cap-dependent) from AUG1 possible initiation from IRES structure (cap-independent) interaction of the U118-A220 hairpin with HDM2 proposed riboswitch-type regulation (Chen et al., 2025)	activates the transcription of genes responsible for apoptosis, cell cycle arrest, and DNA repair the main guardian of the genome/suppressor of tumorigenesis and viral infection development
Δ40p53/ΔNp53/p47	the same transcripts as for p53FL or possible alternative splicing with retained intron 2 (Marcel et al., 2011)	lack of the first transactivation domain (TAD1) possible presence of a sequence from intron 2 partial secondary structure model has been proposed (Figure 1D) (Górska et al., 2013) does not contain the HDM2-binding site/hairpin	alternative translation initiation site AUG2 possible initiation from IRES structure (cap-independent) (Ray et al., 2006) created during ER stress; mechanism unclear	partial loss of transcriptional activation ability can modulate the activity of full-length p53, longer half-life homooligomerization induces G2 (in contrast to p53, which induces G1 arrest) effect on aging
Δ133p53	alternative promoter P2 in intron 4	transcript starts in the middle of the gene → lack of exons encoding transactivation- and proline rich domains partial secondary structure model (Figure 1E) has been proposed (Żydowicz-Machtel et al., 2021)	classic translation initiation from AUG3 possible cap-independent mechanism – *in vitro* studies (Żydowicz-Machtel et al., 2021)	may modulate the activity of full-length p53 acts dominant-negative to p53α promotes cell proliferation and survival, despite possible DNA damage or viral infection possible pro-viral and pro-tumorigenic effect; associated with cellular senescence
Δ160p53	alternative promoter P2 (same mRNA as for Δ133p53)	even shorter N-terminal variant than Δ133p53 partial secondary structure model (Figure 1E) has been proposed (Żydowicz-Machtel et al., 2021)	alternative translation initiation site AUG4 (López-Iniesta et al., 2022)	may modulate the activity of full-length p53 stronger dominant-negative effect; associated with cancer progression and viral development
p53β	transcription from promoter P1, P0 or P’; and alternative splicing in intron 9	replacement of the C-terminus with a short RNA sequence	classic translation initiation (cap-dependent) from AUG1	increases pro-apoptotic activity may support cellular senescence
p53γ	transcription from promoter P1, P0 or P’; and alternative splicing in intron 9	different C-terminus than β	classic translation initiation (cap-dependent) from AUG1	less known function; suggested role in regulating stress response and differentiation
p53 ψ	transcription from promoter P1, P0 or P’; and alternative splicing in intron 6 (Senturk et al., 2014)	occurrence of a premature termination codon (C-terminal truncation: lack of a critical part of the DNA-binding domain, oligomerization domain, and nuclear localization signal)	classic translation initiation (cap-dependent) from AUG1	does not exhibit transcriptional factor activity (unable to bind to DNA and does not transactivate canonical p53 target genes) leads to the cell acquiring mesenchymal cell characteristics (transition to metastasis) p53Ψ is inherently expressed in tumors, and during tissue injury

To date, at least 13 isoforms of the p53 protein have been identified, differing in their domain content and, consequently, in their location and functions within the cell, but also in their copy numbers and relative proportions within the total cellular p53 pool (Marcel et al., 2011a; Guo et al., 2024; Senturk et al., 2014). These proportions depend on changing conditions and states of the cell, such as stress, cell cycle phase, or age. It has also been observed that the synthesis of individual isoforms in cells depends on tissue type and can be regulated under various conditions of cellular stress (Powell et al., 2008; Bourougaa et al., 2010; Marcel et al., 2011a). Furthermore, studies indicate that p53 protein isoforms may play an important role in regulating p53 activity within the cell (Bourdon et al., 2005).

N-terminal isoforms can be divided into “long” and “short” ones. “Long” are p53FL variants and Δ40p53, starting at AUG1 or 2. “Short” refers to those that start with the AUG 3 or 4: Δ133p53 and Δ160p53. C-terminal isoforms are indicated with suffixes: α, β, γ, or ψ, depending on the region of translation termination (Figure 1B) and are characterized by different subcellular localization and limited oligomerization capacity (Bourdon et al., 2005; Marcel et al., 2011a). In most cells, p53α, Δ133p53, and p53β are mainly found in the cell nucleus, but small amounts can also be detected in the cytoplasm. The p53γ isoform can be located in both the nucleus and the cytoplasm, while the Δ133p53γ isoform occurs only in the cytoplasm.

Δ40p53 lacks a transactivation domain, so its ability to activate gene transcription is severely limited. Since there is no amino terminus to recruit chromatin remodeling factors, Δ40p53 cannot regulate gene expression as effectively as p53FL (Courtois et al., 2002; Powell et al., 2008; Olivares-Illana and Fahraeus, 2010).

Another consequence of the absence of this domain is the loss of protein-protein interactions with HDM2 (human double minute 2 protein), which, in turn, means that this isoform is not ubiquitinated and degraded by the proteasome, unlike full-length p53. As a result, the half-life of Δ40p53 in the cell is significantly longer, at 9.5 h (Marcel and Hainaut, 2009). Similar to p53FL, the Δ40p53 isoform has a DBD domain and an oligomerization domain, thanks to which it can form part of p53 tetramers and disrupt their action as a transcription factor.

### Different transcriptional promoters

Currently, at least five promoter sites are known: P0, P1, and P1′ located in exon 1, and Pin and P2 located in introns 1 and 4, respectively (Figure 1A). The alternative use of these sites results in the generation of RNA transcripts of different lengths. In the case of such variants of 5′ mRNA for α, β, and γ p53, the lengths of their 5′UTR regions will vary from 248 nucleotides (P0) to 190 nt (P′) to 144 nt (P1) (Tuck and Crawford, 1989; Strudwick et al., 2003). Little is known about the transcript produced from the P^in^ promoter. However, it has been noted that in healthy tissues, the p53 protein is synthesized from the P0 or P′ promoter (Strudwick et al., 2003), and in cancerous tissues, from P1. In this case, the different lengths of the 5′UTR may be related to the condition of the cells in which p53 is expressed, which can be linked to its structure and the content of sites of interaction with proteins and regulatory RNA molecules.

### Alternative splicing (pre-mRNA level)

The C-terminal isoforms of p53 (α, β, and γ) result from alternative splicing of intron 9. They do not contain an oligomerization domain, an NLS (nuclear localizing sequence), or a C-terminal domain. The newest isoform of the p53 protein identified to date, p53 ψ, discovered in 2014, is synthesized from an mRNA template generated by alternative splicing of intron 6 and may influence the transition of the cell to metastasis (Figure 1; Table 1; Senturk et al., 2014). There is also a theory of alternative splicing of intron 2, which is involved in the creation of the Δ40p53 isoform and is connected with the presence of G-Q structure in pre-mRNA: In recent years, it has been proposed that higher-order RNA structures, such as G-quadruplexes (G-Qs), may play a crucial role in regulating splicing (Marcel et al., 2011b). This structure forms due to the high guanosine content, which interacts to form a four-stranded structure. It has been observed that intron 3 of the TP53 gene contains a guanine-rich sequence that can also form a G-Q at the pre-mRNA level. The formed G-quadruplex motif may stimulate the complete excision of intron 2. In contrast, mutations that linearize this motif result in the retention of intron 2 in the mature mRNA, which, in turn, stimulates the translation/synthesis of Δ40p53 (Marcel et al., 2011b). The G-Q mutation, therefore, increases the synthesis of this isoform at the expense of p53FL.

### Different translation initiation sites and RNA structure contribution

It is not precisely known how many mature p53 mRNA molecules are produced from the template on which its isoforms are synthesized. Due to the presence of specific RNA structures, more than one isoform can be produced from one type of mRNA. The opposite situation is also possible, in which the same isoform can be produced from several mRNA variants that differ, for example, in the use of a different promoter. For example, the main isoform, p53α, can be produced from mRNA variants starting at P0, P1, or P′, and the Δ40p53 isoform can be produced from the same mRNA as the main isoform (thanks to the phenomenon of leaky scanning or the IRES structure), theoretically from mRNA starting at P^in^ (in intron 1), as well as from mRNA with intron 2 preserved. An important factor influencing the frequency or level of translation of individual p53 isoforms is the secondary structure of the regulatory regions contained in mRNA molecules, especially in the 5′ non-coding region, often co-created by the sequence of both the non-coding and coding parts of a given isoform.

## Structure of the 5′ terminal region of the p53 mRNA, responsible for initiating its translation

The biosynthesis of p53 protein and its isoforms is strictly controlled by the presence of stable secondary structure elements located within the 5′ non-coding region of p53 mRNA (Vilborg et al., 2010; Błaszczyk and Ciesiołka, 2011; Gorska et al., 2013). The length of the 5′UTR and the secondary structure of this region significantly impact the migration of the 43S PIC complex, which binds to mRNA to identify the AUG initiation codon (Leppek et al., 2018). 5′ UTR regions that are highly structured and thermodynamically stable can cause delays in scanning by the ribosomal complex and lead to a decrease in translation efficiency. Additionally, the initiation of p53 isoform translation may also be controlled via IRES elements present in the 5′ terminal region of p53 mRNA (Ray et al., 2006; Candeias et al., 2006). Furthermore, the high structural complexity of the 5′ UTR region also influences its interaction with RNA-binding proteins (RBPs), which can either enhance or suppress translation initiation (Takagi et al., 2005; Leppek et al., 2018; Swiatkowska et al., 2016). More below.

In the 5′ terminal regions of p53 mRNA (variants transcribed from P0 and P1), there are two characteristic hairpin-like structural motifs: G56–C169, where the codon AUG1 for the p53 protein is located, and U180–A218, which interacts with the HDM2. Additionally, this region contains an IRES element, which is involved in regulating p53 translation (Figure 1C).

The role of individual elements of the 5′ mRNA structure (Figures 1C,D) in the translation efficiency of the main isoform p53α and Δ40p53α has been described in detail in several studies (Gorska et al., 2013; Swiatkowska et al., 2016; Szpotkowska et al., 2019), and for the translation of shorter isoforms – Δ133p53 and Δ160p53 – in the work of Żydowicz-Machtel et al. (2021). The structural context of the start codon, the length of the UTR, and the presence of key structures that bind the primary regulators of translation of a given mRNA are all significant factors affecting translation efficiency. In the case of p53FL variants, the structure responsible for interactions with HDM2 is the U180–A218 hairpin.

### Structure of the 5′ terminal region of the Δ40p53 isoform mRNA, responsible for initiating its translation

The first well-characterized isoform of the p53 protein, and the most common, apart from the full-length isoform, was the Δ40p53 isoform, which is produced by alternative translation initiation (Courtois et al., 2002). The synthesis of Δ40p53 occurs from the AUG2 initiation codon located in exon 4 of the TP53 gene, as a result of which the first 39 amino acids shorten this isoform compared to the full-length p53. Importantly, the synthesis of the Δ40p53 isoform under conditions of cellular stress can occur independently of the 5′ cap structure and scanning mechanism, but through the binding of the ribosomes directly at the site of translation initiation using the IRES element (Ray et al., 2006; Candeias et al., 2006).

### Structure of the 5′ terminal region of the C-terminal/“short” isoforms of p53: Δ133p53, and Δ160p53

There is a P2 transcription initiation site within intron 4 of the human TP53 gene. This transcript encodes two further p53 isoforms: Δ133p53, whose translation begins at codon AUG3, and Δ160p53, initiated at codon AUG4 (Bourdon et al., 2005; Marcel et al., 2010) (Figure 1; Table 1). These isoforms are potentially involved in carcinogenesis, but their biological function remains unclear (Arsic et al., 2015; Gadea et al., 2016; Guo et al., 2024), as they inhibit the suppressor activity of p53α. They promote cell proliferation and survival despite potential DNA damage or viral infection and may also exhibit pro-viral and pro-neoplastic effects while simultaneously displaying anti-aging effects.

*In vitro* studies (Żydowicz-Machtel et al., 2021) suggest this possibility (IRES) for Δ133p53, but other reports indicate that it is formed from a separate mRNA starting at the P2 site (Marcel et al., 2010).

The secondary structure of the 5′ terminal region of Δ133p53 mRNA encoding Δ133p53 and Δ160p53 isoforms was determined. Using the SHAPE method and RNA cleavage in the presence of Pb^2+^ ions, the structure was defined, and a model of the secondary structure of the 5′ non-coding region of mRNA was proposed (Żydowicz-Machtel et al., 2021). The 5′ terminus of this region is extensively base-paired with its 3′ part. The first translation initiation codon AUG3 is located in an unusual structural environment, just between two hairpin motifs. Moreover, the second hairpin is a part of a three-hairpin domain that separates the AUG3 and AUG4 codons. The high thermodynamic stability of this domain (ΔG = −29 kcal/mol) and the conservation of nucleotides in this mRNA region suggest that the three-hairpin domain may play an important functional role. Furthermore, an *in vitro* translation study conducted in the presence of a cap analog showed that initiation from the AUG3 codon is independent of the cap structure. Previous reports have indicated the formation of both isoforms, Δ133p53 and, from the same transcript (Bourdon et al., 2005; Marcel et al., 2010). Recently, it has also been noted that another AUG codon, 21 nt downstream, at position Δ169p53, can be used for the biosynthesis of this isoform (López-Iniesta et al., 2022). Functionally, the Δ160p53 isoform can modulate the activity of full-length p53. It can influence DNA repair by interacting with other p53 isoforms or transcription factors, acting as a positive or negative regulator, depending on the cellular context. However, it also acts independently, influencing proliferation and inhibiting the apoptotic cell pathway, which may be important in delaying the aging process. However, it also promotes the survival of cancer cells despite DNA damage (Marcel et al., 2011a; Fujita et al., 2009).

## RNA-RNA and RNA-protein interactions

Recent work suggests that the action of the U180-220A structure, known also as BOX-1, may be a potential riboswitch sensitive to the cascade of signals triggered by DNA damage (Chen et al., 2025). In its inactive, blocked state, the apical loop interacts with the sequence above and is hidden from interaction with the HDM2 protein. In its active state, it is released from this interaction to favor interaction with the protein and enhance translation efficiency. In this work, the mechanism blocking protein binding is a loop-loop interaction between three nt residues of the apical loop of the aforementioned BOX-1 hairpin, with the nt residues of the apical loop of another hairpin 1–40, which in the structure taking into account the 5′UTR sequence corresponds to the side loop of the hairpin-like structure: G56–C169 (Figure 1C – the interaction is marked with blue stripes).

### Long-range interaction between the 5′UTR and 3′UTR

Another interesting structural motif related to the *in trans* interaction regulating translation is the long-range interaction between the 5′UTR and 3′UTR of the p53FL transcript, first proposed by Chen and Kastan (2010) and Chen et al., (2012) and recently elaborated by Kiliszek et al. (2023): In unstressed cells, NCL (nucleolin) promotes the base-pairing of the 5′UTR and 3′UTR of p53 mRNA, starting at the bulge and extending to the distal part of the 5′ CS, stabilizing the higher-order RNA–RNA contact. As long as NCL occupies the 5′CS/3′CS double-stranded region, translation of the p53 protein is repressed. Under stress conditions (DNA damage), RPL26 displaces NCL via protein-protein interactions that disrupt NCL-NCL homodimers hereby enhancing translation of the p53 protein (Kiliszek et al., 2023).

### Pairing of the p53 transcript with its antisense, non-coding RNA – WRAPα

An exciting example of p53 level control in the cell is its interaction with its natural antisense transcript, Wrap53α. It has been shown that the RNA transcripts Wrap53α and P1-p53 interact with each other via overlapping, fully complementary sequences several dozen base pairs in length. Exon 1 of Wrap53 mRNA binds to exon 1 of p53 mRNA in the initial 5′UTR sequence. As a consequence of this interaction, increased p53 protein translation has been observed (Mahmoudi, 2009). Depletion of Wrap53 or blockage of Wrap53/p53 RNA hybrids) prevented p53 protein induction and transactivation of p53 target genes in response to DNA damage (Farnebo, 2009). This phenomenon can be explained by the increased stability of mRNA in its hybridized form with WRAP, which allows the paired region to be hidden from degradation, thereby facilitating more efficient translation.

### Influence of PERK on structural changes in p53 mRNA

It has recently been shown that PERK kinase is a key factor responsible for the structural change in human p53 mRNA, which is crucial for the alternative initiation of p47 (Δ40p53) isoform translation during endoplasmic reticulum (ER) stress. However, the direct interaction between PERK and p53 mRNA has not been demonstrated, suggesting that the mechanism mediating these changes in mRNA structure remains unknown. The study employed a dominant-negative PERK mutant (PERKAC), whose expression reverses the structural changes in p53 mRNA induced by ER stress, confirming that these changes are dependent on PERK activity. However, eIF2α phosphorylation, often associated with PERK activity, is not involved in this process. Additionally, attempts to silence trans-activating factors such as hnRNP C1/C2 and PTB have shown that these proteins alone are not responsible for these structural changes (Chen et al., 2025). The relationship of this kinase to a specific factor (e.g., RNA chaperone type) responsible for remodeling the RNA structure in response to PERK activity remains unknown. It is the subject of further research (Chen et al., 2025).

## Discussion

One explanation for the claim that p53 protein isoforms may be important in regulating p53 activity in the cell (Bourdon et al., 2005) is the tetramer theory. According to this theory, the p53 protein acts as a transcription factor in the form of a tetramer, which can include different types of p53 isoforms, as long as they contain an oligomerization domain and are located in the nucleus. The composition of the tetramer, therefore, depends on the availability of individual isoforms at a given time and place. The way in which this factor acts depends on which isoforms form the tetramer, sometimes contrary to what we would expect from the main protein isoform. For example, suppose the tetramer is formed by isoforms that lack a transactivation domain. In that case, it will not act as a transcription factor, and if the tetramer is formed by isoforms lacking the HDM2 protein-binding domain, it will not undergo ubiquitination. Its duration and action in the cell will be prolonged. In this case, depending on the composition of the p53 isoform pool in the cell, p53 tetramers will act differently.

Most often, when N-terminal isoforms lacking the transactivation domain form a p53 tetramer, they activate other genes less effectively or not at all, which may result in cell cycle arrest, inhibition of apoptosis, and enhanced proliferation, regardless of whether DNA mutations or viral infection have occurred. It is easy to imagine the future fate of the cell and the organism when p53 function is impaired: unchecked cancer development, viral infection, or accelerated cellular aging. Sometimes, however, the action of isoforms can be beneficial. For example, when the alarm signal read by p53 was false (i.e., it came from a situation that is not an actual threat). In case of C-terminal isoforms that lack the oligomerization domain, they will not participate in tetramer formation, but their action will occur at a different level and in a different way.

The mechanisms responsible for generating over a dozen isoforms from a single gene comprise the use of different transcriptional promoters (DNA level), alternative splicing (pre-mRNA level), and different translation initiation sites (mRNA level). *In vitro* studies have demonstrated that translation initiation at different sites on full-length p53FL mRNA is possible due to specific RNA structures, and that these structures are also responsible for the rate and efficiency of target protein isoform formation.

Unfortunately, some studies of the structure and role of RNA transcripts and their fragments (not only in the case of p53) do not take into account the presence of sequences upstream the start codon, which is a grave mistake, as the presence of an adjacent RNA sequence, additionally rich in G and C residues, may be of great importance in shaping the structure of the fragment under study. The same applies to studies of 3′UTRs. If we do not account for adjacent coding sequences, we may obtain false results. This should also be kept in mind when studying reporter transcripts, i.e., those where the coding region contains a reporter gene sequence that can alter the structural context of the studied UTR fragment.
